# Systematic review and meta-analysis of Chinese herbal formula Tongxie Yaofang for diarrhea-predominant irritable bowel syndrome: Evidence for clinical practice and future trials

**DOI:** 10.3389/fphar.2022.904657

**Published:** 2022-08-25

**Authors:** Shi-Bing Liang, Hui-Juan Cao, Ling-Yao Kong, Jia-Li Wei, Nicola Robinson, Si-Hong Yang, Si-Jia Zhu, Yu-Qi Li, Yu-Tong Fei, Mei Han, Jian-Ping Liu

**Affiliations:** ^1^ Centre for Evidence-Based Chinese Medicine, Beijing University of Chinese Medicine, Beijing, China; ^2^ School of Traditional Chinese Medicine, Beijing University of Chinese Medicine, Beijing, China; ^3^ The Third Affiliated Hospital of Beijing University of Chinese Medicine, Beijing, China; ^4^ Institute of Health and Social Care, London South Bank University, London, United Kingdom; ^5^ China Center for Evidence Based Traditional Chinese Medicine, China Academy of Chinese Medical Sciences, Beijing, China; ^6^ Institute of Basic Research in Clinical Medicine, China Academy of Chinese Medical Sciences, Beijing, China; ^7^ The National Research Center in Complementary and Alternative Medicine (NAFKAM), Department of Community Medicine, Faculty of Health Science, UiT the Arctic University of Tromsø, Tromsø, Norway

**Keywords:** tongxie yaofang, Chinese herbal formula, irritable bowel syndrome, randomized controlled trial, systematic review, meta-analysis

## Abstract

**Introduction:** Diarrhea-predominant irritable bowel syndrome (IBS-D) significantly decreases the quality of life of patients and their families, and affects patients’ mental health. No specific western medications are available. Ancient classical Chinese medical texts have recognized Tongxie Yaofang (TXYF) as a therapy for diarrhea which is widely used in clinical practice. Standard TXYF prescription (S-TXYF) is composed of four herbal medicines: *Atractylodes macrocephala* Koidz. [Asteraceae; *Rhizoma Atractylodis Macrocephalae*.], Paeonia lactiflora Pall. [Ranunculaceae; Paeoniae Radix Alba], Citrus × aurantium L. [Rutaceae; Citri Reticulatae Pericarpium] and Saposhnikovia divaricata (Turcz. ex Ledeb.) Schischk. [Umbelliferae*; Saposhnikoviae Radix*]. This review aimed to evaluate the therapeutic effects and safety of S-TXYF for IBS-D.

**Methods:** Eight English and Chinese electronic databases were searched from their inception to 25 December 2021 for randomized controlled trials (RCTs) comparing S-TXYF with placebo, western medications or no treatment for IBS-D. The primary outcome was the global improvement of IBS-D symptoms. Data were analyzed using Cochrane’s Revman 5.4 software. Evidence certainty was assessed using the online GRADEpro tool for the primary outcome.

**Results:** Eleven RCTs involving 985 adults with IBS-D were included. For global improvement of symptoms, S-TXYF was superior to western medication and placebo (moderate evidence by GRADE). Regarding the improvement of stool consistency, stool frequency and abdominal pain, S-TXYF was significantly effective than placebo. In addition, S-TXYF was superior to western medication on improving the quality of life and relieving anxiety. Six trials reported adverse events: five of them reported (non-serious) adverse events occurred in both groups, and one trial reported that 3 cases with adverse events (constipation, elevation in liver-enzyme, nausea) occurred in S-TXYF group and 3 cases with adverse events (abdominal distension, nausea) occurred in placebo group.

**Conclusion:** Although current results showed that S-TXYF may have potential to treat IBS-D and its use appears to be safe, no a clear and confirmed conclusion can be drawn from our review as the overall inadequate design of the included trials reviewed. So more rigorous trials are warranted to establish confirmed evidence on its benefits and safety.

## 1 Introduction

Irritable bowel syndrome (IBS) is a functional bowel disorder with a high population prevalence, and is characterized by chronic, recurrent, abdominal pain and discomfort, and altered bowel habits that occur in the absence of other organic gastrointestinal (GI) diseases (Mearin et al., 2016; Defrees and Bailey, 2017; Ford et al., 2017). Researches (Lovell and Ford, 2012; Oka et al., 2020) has demonstrated that the global prevalence of IBS can reach 11%, but may vary considerably (from less than 1% to more than 25%), according to geographic region, diagnostic criteria used to define IBS, minimum symptom duration required, age, and gender. In any case, IBS significantly decreases the quality of life of patients, their partners and caregivers, and affects psychological health status of patients (Hungin et al., 2005; Ford et al., 2014). IBS can be sub-classified into four types including constipation type (IBS-C), diarrhea type (IBS-D), mixed type (IBS-M) and undefined type, according to the predominant stool pattern according to Rome VI criteria (Ford et al., 2017). Researches on the global prevalence of IBS documented a pooled prevalence of 23.4–40% for IBS-D (Lovell and Ford, 2012; Bellini et al., 2014). The underlying pathogenesis of IBS is considered to be complex and still incompletely known and the precise molecular pathophysiology is far from understood (Saito and Talley, 2008; Ford et al., 2014), and no specific therapeutic medications have been found.

**FIGURE 1:**
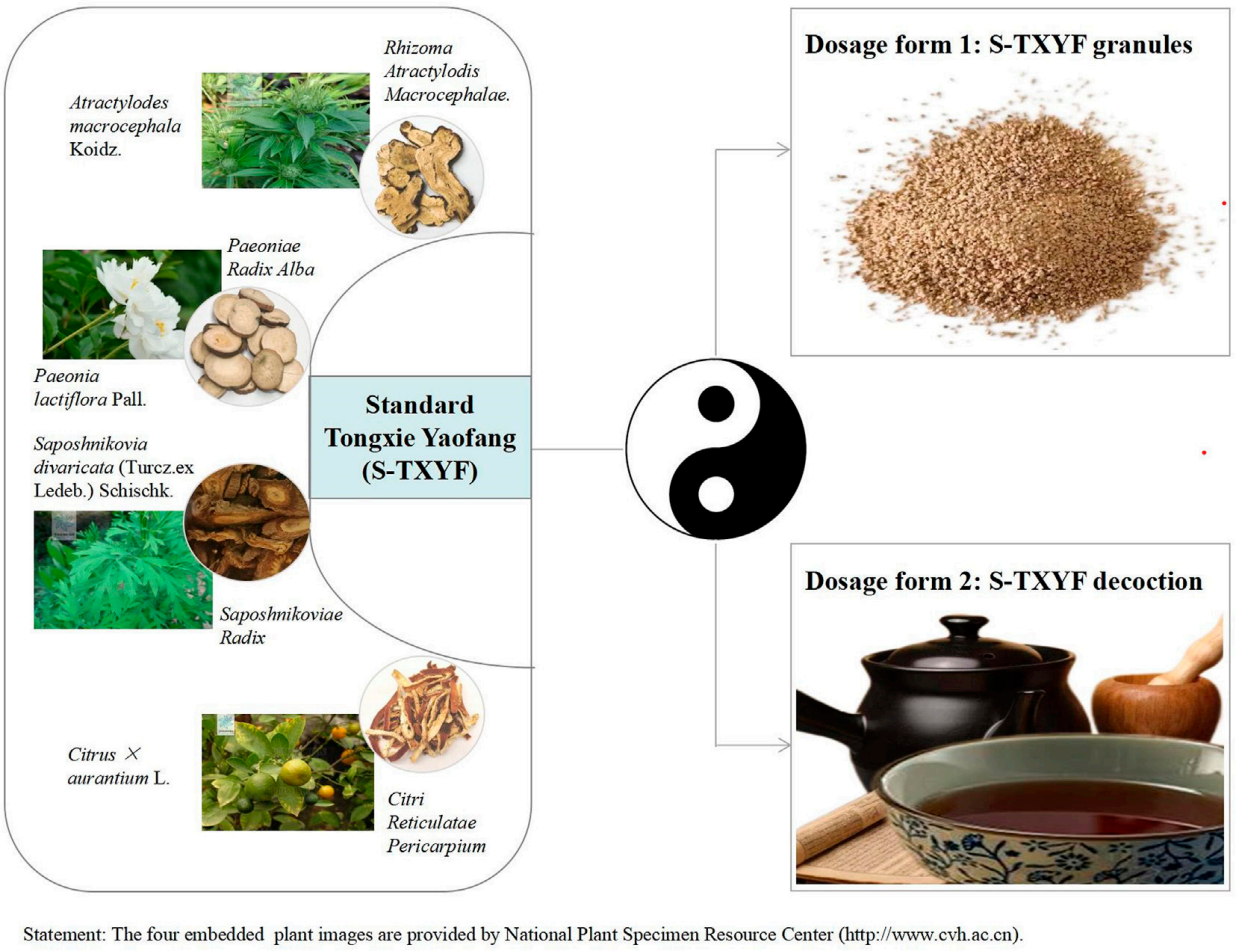
The composition and clinical dosage forms of standard Tongxie Yaofang.

In traditional Chinese medicine (TCM), IBS-D belongs to a specific category of diarrhea. A high-quality randomized controlled trial (RCT) published in JAMA confirmed that Chinese herbal medicine (CHM) is indeed effective for IBS and the author also concluded that these findings support the consideration of further investigation of CHM as a treatment option for IBS (Bensoussan et al., 1998). Tongxie Yaofang (TXYF), one of the most commonly used Chinese herbal formula, was first recorded in the Yuan Dynasty (1271-1368) (Dai et al., 2017). Standard TXYF (hereinafter referred to as S-TXYF, here we only consider the types of herbal medicines that make up TXYF, and does not consider the dose of a single herbal medicine) has been used for centuries to treat diarrhea and is composed of four Chinese herbal medicines: *Atractylodes macrocephala* Koidz. [Asteraceae; Rhizoma Atractylodis Macrocephalae.], Paeonia lactiflora Pall. [Ranunculaceae; Paeoniae Radix Alba], Citrus × aurantium L. [Rutaceae; Citri Reticulatae Pericarpium] and Saposhnikovia divaricata (Turcz. ex Ledeb.) Schischk. [Umbelliferae*; Saposhnikoviae Radix*] (Liang et al., 2022). In clinical practice it is prescribed either as S-TXYF granules or S-TXYF decoction. Figure 1 shows the composition and clinical dosage forms of S-TXYF.

Previous research (Sun et al., 2020) exploring the mechanism of S-TXYF for IBS-D based on the network pharmacology found that the mechanism of TXYF to treat IBS-D is characterized by multiple active components (alcohols, lactones, glycosides, flavonoids etc.), multiple core targets (VEGFA, AR, OPRM1, PGR and ESR1), and multiple pathways (metabolism, G protein-coupled receptor mediated signal transduction, immune system function and nerve growth factor mediated signal transduction). Through the above mechanisms of action, S-TXYF may reduce visceral hypersensitivity, improve the pain threshold of viscera, and reduce gastrointestinal irritation, thereby relieving the symptoms of IBS-D e.g. abdominal pain and diarrhea, and promoting the cure of IBS-D. Moreover, extensive animal experiments (Peng et al., 2020; Zhang et al., 2020; Li et al., 2021) illustrated that S-TXYF can regulate the gut microbiota. In addition, another net-work pharmacological research (Man et al., 2022) presented that S-TXYF may have some common mechanisms in the treatment of IBS and depression, such as oxidative stress, immune regulation, regulating inflammatory response, promoting apoptosis and endocrine metabolism. But in any case, what we expect is that S-TXYF can be effective and safe when used in humans. So the question that should to be answered and clarified is that whether S-TXYF is truly effective and safe to treat IBS-D in clinical practice. To our knowledge, although some clinical trials of S-TXYF on IBS-D have been published, no relevant systematic review and meta-analysis based on RCTs has been published. And we don’t know the quality of those trials that have been published. Therefore, our research aims to evaluate the therapeutic effects and safety of S-TXYF for IBS-D so that to provide a high-quality evidence for future clinical practice and trials.

## 2 Methods

### 2.1 Eligibility criteria

RCTs comparing S-TXYF with western medication, placebo or no treatment in adults with IBS-D were included. IBS-D was diagnosed according to a clear criteria such as the Rome criteria. S-TXYF trials were eligible for inclusion without limitations to dosage, formulation or treatment duration. At least one of the following outcomes had to be presented for evaluation by the included trial: (a) global improvement of IBS-D symptoms, measured by a validated scale or efficacy evaluation criteria, such as IBS severity scoring system (IBS-SSS) (Francis et al., 1997), or other scales or criteria with a clear description; (b) stool consistency; (c) stool frequency; (d) abdominal pain; (e) quality of life measured by validated tools, such as the 36-MOS item short-from health survey (SF-36) (Alonso et al., 1995) or the scale of IBS-quality of life (IBS-QOL) (Patrick et al., 1998); (f) anxiety measured using the self-rating anxiety scale (SAS) (Zung, 1971) or other validated scales; (g) depression measured using the self-rating depression scale (SDS) (Zung, 1965) or other validated scales; (h) recurrence rate at the end of follow-up and (i) (Severe) adverse events. In our review, global improvement of IBS-D symptoms was the primary outcome, and the remainders were secondary outcomes. Regarding the time period, all lengths of treatment time and duration of follow-up were eligible. For outcomes reported at multiple time points, we used the time point at the end of the treatment and the longest reported follow-up time point.

Exclusion criteria: (a) the full text of publication could not be obtained; (b) duplicated articles; (c) trial protocols; (d) conference articles.

### 2.2 Search strategy

PubMed, the Cochrane Library, Embase, Web of Science, SinoMed, the Chinese National Knowledge Infrastructure Databases (CNKI), the Chongqing Chinese Science and Technology Journal Database (VIP) and Wanfang Database were searched from their inception to 25 December 2021.

The subject/Mesh terms used for the searches were "Tongxie Yaofang" OR "Tong Xie Yao Fang" OR "Tong-Xie-Yao-Fang" OR "TXYF" combined with "Irritable bowel syndrome" OR "IBS", and adjusted for use in the different databases. The detailed search strategies for all databases are listed in the Supplementary Appendix Table S1.

### 2.3 Study selection and data extraction

Two authors (SBL, LYK) independently screened the titles, abstracts and full reports of the retrieval records, and selected the publications of eligible studies according to the eligibility criteria. Then, another two authors (LYK, JLW) independently extracted the data based on the pre-designed form, including the author’s information, characteristics of participants, details of interventions and controls, outcomes, and information relevant to the study design.

### 2.4 Risk of bias assessment

The risk of bias (ROB) for the primary outcome of each included trial was assessed using the Cochrane risk of bias tool 2.0 (ROB 2.0) (Sterne et al., 2019) by two authors (SBL, SJZ) independently. Inconsistencies were discussed with a third author (JPL).

The ROB 2.0 consists of the following domains: (a) randomization process; (b) deviations from the intended interventions; (c) missing outcome data; (d) measurement of the outcome; and (e) selection of the reported result. Each domain was judged as low risk of bias, high risk of bias or some concerns.

Finally, an overall assessment for each trial was developed based on the results of the above five domains in each trial. It was also judged as low risk of bias, high risk of bias or some concerns.

### 2.5 Data synthesis

The data were analyzed by Review Manager 5.4 (Revman 5.4, Copenhagen: The Nordic Cochrane Centre, The Cochrane Collaboration) software (Review Manager, 2020). Risk ratio (RR) with its 95% confidence intervals (CI) as well as the number needed to treat (NNT) were applied to dichotomous outcomes, and mean difference (MD) with its 95% CI were applied to continuous outcomes.

The random-effects model was used for meta-analysis considering potential sources of clinical heterogeneity. The value of *I* ^2^ judges the size of heterogeneity among the included trials in each meta-analysis. The smaller the value, the smaller the statistical heterogeneity (Higgins et al., 2020). If the *I* ^2^ ˃ 50%, the data accuracy was checked firstly. If the data was accurate and appropriate, subgroup analysis based on important factors of baseline, interventions and comparators and/or sensitivity analysis based on methodological quality would be conducted to explore the source of heterogeneity and the results interpreted carefully.

Subgroup analyses would be conducted for the primary outcome, if appropriate: 1) based on the treatment duration, to explore whether patients get better therapeutic effects with a longer treatment duration; 2) based on the dosage forms of S-TXYF, to explore whether dosage form has an impact on the therapeutic effects; 3) based on the different medications of comparator, to explore whether there will be significant differences between S-TXYF and different western medications.

Sensitivity analysis was also used to assess robustness of results between fixed-effects and random-effects analysis.

Evidence certainty for the primary outcome was assessed using the GRADE (Grading of Recommendations Assessment, Development and Evaluation criteria) approach (Guyatt et al., 2008). And, besides the above analyses, a funnel plot would be applied to explore the possibility of publication bias, if ten or more trials were enrolled in a single meta-analysis (Egger et al., 1997).

## 3 Results

### 3.1 Search results

**FIGURE 2:**
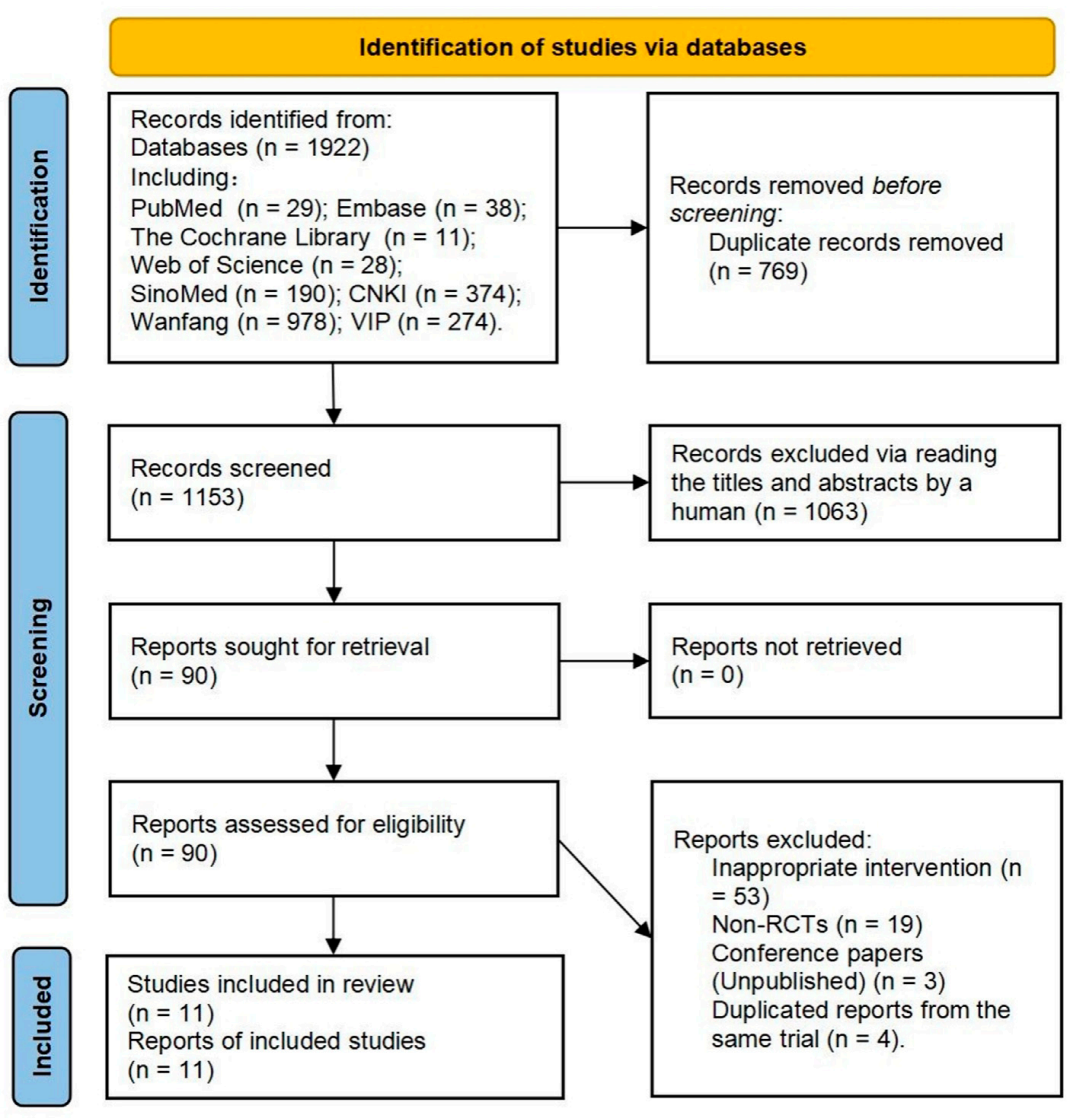
The flow diagram of study retrieval and selection.

In total, 1922 records were retrieved. Finally, 11 reports (representing 11 trials) (Li, 2006; Pan et al., 2009; Kong et al., 2010; Lin, 2012; Tang, 2017; Zhang et al., 2017; Chen et al., 2018; Lin, 2019; Hao and Shi, 2020; Wang et al., 2020; Yao et al., 2020) were included. Figure 2 provides the flow diagram of the study retrieval and selection.

### 3.2 Characteristics of the included trials

The trials were all conducted in China and published between 2006 and 2020. Of which, five trials (Pan et al., 2009; Kong et al., 2010; Zhang et al., 2017; Chen et al., 2018; Yao et al., 2020) were two-armed trials, and the remaining six trials (Li, 2006; Lin, 2012; Tang, 2017; Lin, 2019; Hao and Shi, 2020; Wang et al., 2020) were three-armed trials (only two arms that met the inclusion criteria were used for analysis in each trial). A total of 985 participants were included for data analysis, of which 505 were in the S-TXYF group. The ratio of male to female was 1:1.2 based on all the trials reporting this information. All the trials used S-TXYF granules (Li, 2006; Pan et al., 2009; Kong et al., 2010; Lin, 2012; Chen et al., 2018; Wang et al., 2020; Yao et al., 2020) or S-TXYF decoction (Tang, 2017; Zhang et al., 2017; Lin, 2019; Hao and Shi, 2020) as the experimental intervention. The dose ratio of the four herbal medicines (*Rhizoma Atractylodis Macrocephalae., Paeoniae Radix Alba, Citri Reticulatae Pericarpium* and *Saposhnikoviae Radix*) or the four herbal medicines’ extract as granules in different trials was not uniform. No explanation for the choice of the dose ratio was given in all trials. The detailed information of the herbal medicine (extract) dose from each included trials are presented in Table 1. The control interventions include pinaverium bromide tablets (Li, 2006; Kong et al., 2010; Lin, 2012; Zhang et al., 2017; Lin, 2019; Hao and Shi, 2020; Wang et al., 2020; Yao et al., 2020), Trimebutine Maleate (Tang, 2017), Miyarisam (Pan et al., 2009) and S-TXYF placebo granules (Chen et al., 2018). Treatment duration for all included trials varied from 4 to 8 weeks (30 days or 1 month). Table 1 shows the characteristics of the included 11 trials.

**TABLE 1:** Characteristics of the included 11 randomized controlled trials.

Study ID	Diagnostic criteria	Sample size (M/F)		Age (years old)		Course of IBS-D		Experimental intervention	Comparator	Treatment duration	Outcomes
		T	C	T	C	T	C				
Kong et al. (2010)	Rome III	28/22	23/27	49.23 ± 12.45	48.46 ± 14.65	5.33 ± 4.62y	5.47 ± 4.53y	S-TXYF granules (no dose information of 4 herbal medicines (or extract)), Jiangyin Tianjiang Pharmaceutical Co., Ltd, 1 bag (^★^10 g) twice daily	Pinaverium bromide, 50 mg thrice daily	4 weeks	①②④⑤
Zhang et al. (2017)	Rome III	14/16	16/14	36.7 ± 9.5	36.1 ± 8.2	4.2 ± 1.1y	4.5 ± 1.4y	S-TXYF decoction (^☆^ *Rhizoma Atractylodis Macrocephalae.* 25g, *Paeoniae Radix Alba* 20g, *Citri Reticulatae Pericarpium* 18g, *Saposhnikoviae Radix* 10g; make these 4 herbal medicines into 300 ml liquid), 150 ml twice daily	Pinaverium bromide, 50 mg thrice daily	1 month	①③④⑨
Yao et al. (2020)	Rome Ⅳ	28/30	25/33	34.5 ± 6.7	33.9 ± 5.3	1.0-5.5years (2.3 ± 1.2)y	1.0-5.0years (2.5 ± 1.3)y	S-TXYF granules (^▼^30 g:20 g:15 g:10 g), Sichuan new green Pharmaceutical Co., Ltd, thrice daily	Pinaverium bromide, 50 mg thrice daily	4 weeks	①⑤⑥⑦⑨
Hao and Shi, (2020)	Rome III	15/27	17/25	39 ± 9 (33-57)	37 ± 7 (33-58)	24 ± 7 m (17-31)m	24 ± 8 m (15-29)m	S-TXYF decoction (^☆^ *Rhizoma Atractylodis Macrocephalae.* 30g, *Paeoniae Radix Alba* 20g, *Citri Reticulatae Pericarpium* 15g, *Saposhnikoviae Radix* 10g; make these 4 herbal medicines into 300 ml liquid), 150 ml twice daily	Pinaverium bromide, 50 mg thrice daily	8 weeks	①
Wang et al. (2020)	Rome Ⅳ	20/21	22/19	29 ± 6 (24-40)	29 ± 5 (26-45)	3.5 ± 1.4 years (1.5-5.5)y	3.6 ± 1.3years (2.0-4.5)y	S-TXYF granules (^▼^30 g:20 g:15 g:10 g), Sichuan new green Pharmaceutical Co., Ltd, thrice daily	Pinaverium bromide, 50 mg thrice daily	4 weeks	①
Tang, (2017)	Rome III	10/20	11/18	44.96 ± 7.12	45.54 ± 6.86	5.02 ± 3.29y	4.88 ± 3.44y	S-TXYF decoction (^☆^ *Rhizoma Atractylodis Macrocephalae.* 20g, *Paeoniae Radix Alba* 20g, *Citri Reticulatae Pericarpium* 12g, *Saposhnikoviae Radix* 15g; make these 4 herbal medicines into 200 ml liquid), 100 ml twice daily	Trimebutine Maleate, 0.2 g thrice daily	8 weeks	①⑤⑥⑦⑨
Li, (2006)	Rome II	11/15	19/23	39.2 (19-64)	39.3 (18-64)	3.92years (1-11)y	4.04years (1-10)y	S-TXYF granules (no dose information of 4 herbal medicines (or extract)), 1 bag (^★^10 g) twice daily	Pinaverium bromide, 50 mg thrice daily	30 days	①⑨
Lin, (2012)	Rome III	16/19	12/23	40.60 ± 9.82	39.51 ± 11.33	NR	NR	S-TXYF granules (no dose information of 4 herbal medicines (or extract)), Jiangyin Tianjiang Pharmaceutical Co., Ltd, 1 bag (^★^10 g) twice daily	Pinaverium bromide, 50 mg thrice daily	4 weeks	①②③④
Lin, (2019)	Rome Ⅳ	16/17	17/16	41.7 ± 20.8	40.6 ± 19.0	NR	NR	S-TXYF decoction (^☆^ *Rhizoma Atractylodis Macrocephalae.* 15g, *Paeoniae Radix Alba* 12g, *Citri Reticulatae Pericarpium* 6g, *Saposhnikoviae Radix* 10g; make these 4 herbal medicines into 400 ml liquid), 200 ml twice daily	Pinaverium bromide, 50 mg thrice daily	4 weeks	①⑨
Pan et al. (2009)	Rome III	33/47	17/23	39.2 ± 13.4	37.5 ± 15.6	6.3 ± 4.6y	5.9 ± 4.5y	S-TXYF granules (^★^extract of *Rhizoma Atractylodis Macrocephalae.*15g, extract of *Paeoniae Radix Alba* 12g, extract of *Citri Reticulatae Pericarpium* 6g, extract of *Saposhnikoviae Radix* 8 g), Jiangyin Tianjiang Pharmaceutical Co., Ltd, 1 bag (^★^41 g) twice daily	Miyarisam, 2 tablets thrice daily	4 weeks	①②④
Chen et al. (2018)	Rome III	41/39	31/49	35.4 ± 10.7	32.7 ± 8.2	4.9 ± 1.6y	5.4 ± 1.5y	S-TXYF granules (Participants orally administrated ^★^25.4 g of S-TXYF granules (provided by Sichuan New Green Pharmaceutical St, Sichuan, China) thrice daily. The S-TXYF granules consisted of 4 herbal medicines’ extract (^★^extract of *Rhizoma Atractylodis Macrocephalae.*10g, extract of *Paeoniae Radix Alba* 6.7g, extract of *Citri Reticulatae Pericarpium* 5g, extract of *Saposhnikoviae Radix* 3.7 g)	S-TXYF placebo granules (provided by Sichuan New Green Pharmaceutical St, Sichuan, China), 25.4 g thrice daily. It was with the same appearance as the S-TXYF granules, and it was made with a mixture of starch, lactose (<1% by weight), food colourants, and bitterants	4 weeks	①②③④⑨

^☆^The dose of the four Chinese herbal medicines.

^★^The dose of S-TXYF, granules or the four herbal medicines’ extract.

^▼^No a clear statement on the forms of Chinese herbal medicine or its extract in the trial’s reporting. The Chinese herbal medicines (or extract) corresponding to each dose are *Rhizoma Atractylodis Macrocephalae., paeoniae radix alba, Citri Reticulatae Pericarpium* and *Saposhnikoviae Radix*, respectively.

S- TXYF is a prescription composed of four herbs including *Rhizoma Atractylodis Macrocephalae., Paeoniae Radix Alba, Citri Reticulatae Pericarpium* and *Saposhnikoviae Radix.* [No single herbal dosage considerations in here.]

S-TXYF, Standard Tongxie Yaofang; M, male; F, female; T, treatment group; C, control group; IBS-D, diarrhea-predominant irritable bowel syndrome; NR, not reported; y, years; m, months.

①Global improvement of IBS-D symptoms; ②Stool consistency; ③Stool frequency; ④Abdominal pain; ⑤Quality of life; ⑥Anxiety; ⑦Depression; ⑧Recurrence rate; ⑨(Severe) Adverse events.

### 3.3 Risk of bias of included trials

**FIGURE 3:**
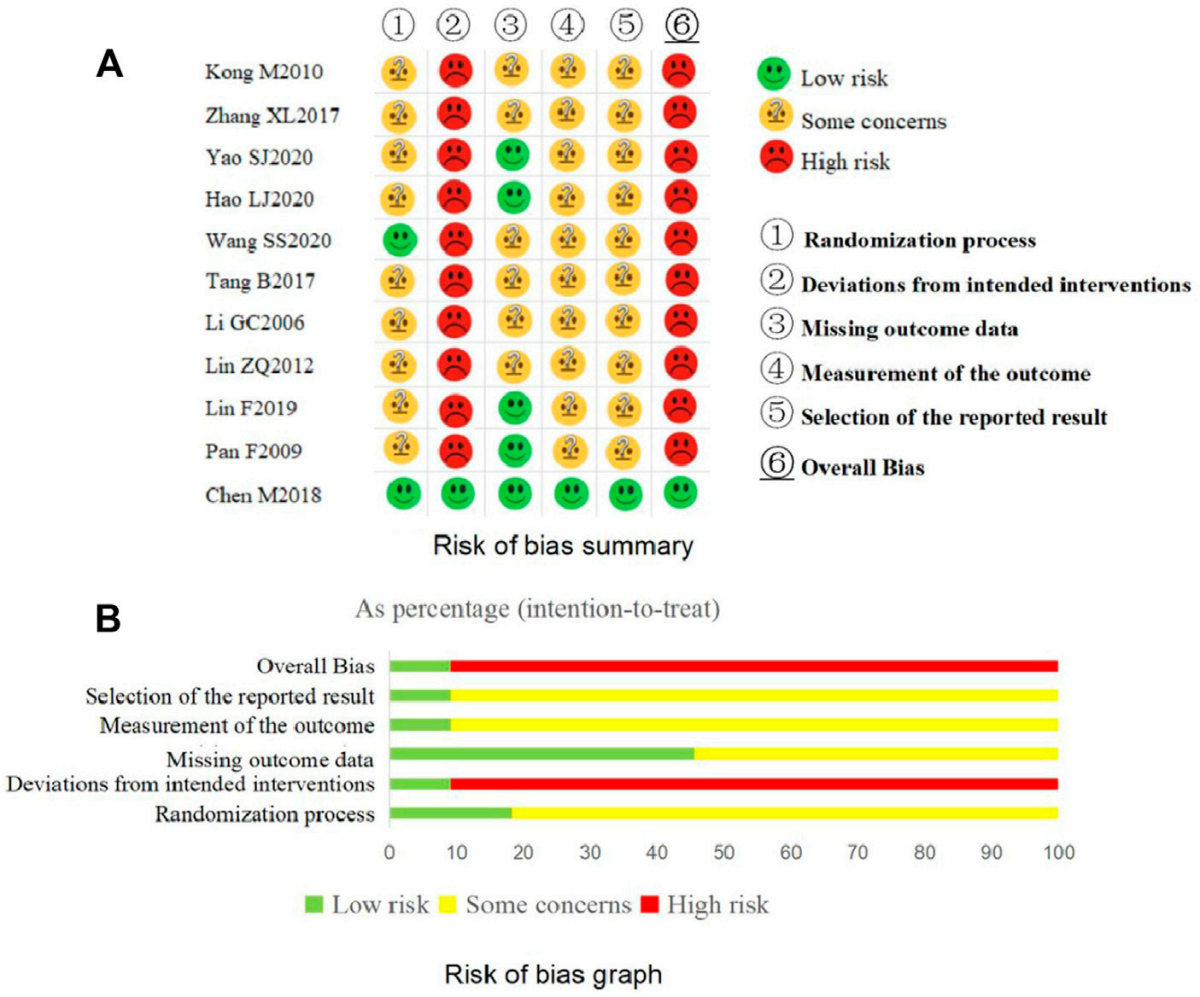
Risk of bias of the included 11 randomized controlled trials.

The overview results for the risk of bias assessment for all included trials are shown in Figure 3.

#### 3.3.1 The risk of bias for each domain of the included trials

The results of the risk of bias assessment for each domain of the included trials are shown in Figure 3.

(a) Domain 1: Randomization process

Three trials (Li, 2006; Pan et al., 2009; Tang, 2017) only mentioned “random” without further clarification and the remaining eight trials (Kong et al., 2010; Lin, 2012; Zhang et al., 2017; Chen et al., 2018; Lin, 2019; Hao and Shi, 2020; Wang et al., 2020; Yao et al., 2020) declared the detailed methods of random sequence generation. The baseline data were comparable for each included trial. Among the included trials, one trial (Wang et al., 2020) used sealed, opaque envelopes as allocation concealment and one trial (Chen et al., 2018) used the central random method. If allocation concealment was not implemented, there is reason to suspect that the enrolling investigator or the participant had knowledge of the forthcoming allocation (Sterne et al., 2019). Considering the above, two trials (Chen et al., 2018; Wang et al., 2020) were judged as “low risk of bias” for this domain and the other trials were judged as “some concerns”.

(b) Domain 2: Deviations from intended interventions

Judged in the light of the interventions, all trials except one (Chen et al., 2018) failed to carry out blinding for participants and clinicians delivering the interventions. These trials also did not report whether deviations arose because of the trial context and the information to assess if the analysis was appropriate was insufficient. Taking the above into consideration, one trial (Chen et al., 2018) was judged as having “low risk of bias” and the other 10 trials were “high risk of bias” in this domain.

(c) Domain 3: Missing outcome data

Complete outcome data available, or data of few participants were missed but appropriate statistical analysis methods such as ITT were used to deal with the missing data, five trials (Pan et al., 2009; Chen et al., 2018; Lin, 2019; Hao and Shi, 2020; Yao et al., 2020) were judged as “low risk of bias” for this domain. The other six trials were considered as having “some concerns” due to no information about the integrity of the outcome data.

(d) Domain 4: Measurement of the outcome

One trial (Chen et al., 2018) conducted blinding for the outcome assessors and statistical analysts. The remaining included trials failed to carry out blinding for clinicians delivering the interventions and they did not report whether the outcome assessors were independent of the clinicians. If the outcome assessors and the clinicians were the same, the outcomes (such as the IBS-SSS) that need to be evaluated by the clinicians were likely influenced by clinicians’ awareness of the interventions received by participants. Taking the above into consideration, one trial (Chen et al., 2018) was assessed as “low risk of bias” and the remainders were assessed as having “some concerns” for this domain.

(e) Domain 5: Selection of the reported result

Only one trial (Chen et al., 2018) reported the information about their protocol, so we could not judge whether the remaining 10 trials selectively report on outcomes. Although the 10 trials reported the primary outcome, various assessment tools/criteria exist for the primary outcome measure in clinical, so we could not to judge whether these trials exist selection on the evaluation tools/criteria as no their protocals been found. Based on the above considerations, one trial (Chen et al., 2018) was judged as having “low risk of bias” and 10 trials were judged as having “some concerns” for this domain.

#### 3.3.2 The overall bias of each included trial

The overall bias was assessed as having low risk of bias in one trial (Chen et al., 2018) and high risk of bias in all the other included trials.

### 3.4 Therapeutic effects and safety evaluation

#### 3.4.1 Primary outcome (global improvement of IBS-D symptoms)

All trials reported this outcome measured with a clear evaluation criteria. We summarized and sorted the evaluation criteria used in all the trials, which can be divided into three types: criteria-1, criteria-2 and criteria-3. Criteria-1: Patients were asked to evaluate whether their main symptoms (stool frequency, stool consistency, abdominal pain, abdominal distension or other discomforts) were fully, partly or not improved. To clearly describe the global improvement of IBS-D symptoms, these categories were classified into “response” or “no response” in our review: if the main symptoms were not improved or even aggravated after treatment, it was considered as no response, otherwise response. Criteria-2: It used the scale of irritable bowel syndromes symptom severity score (IBS-SSS). The scale divided the disease severity into four levels: normal (the score <75), mild (75 ≤ the score <175), moderate (175 ≤ the score <300) and severe (the score ≥300). If the disease severity remains at the original level or changes to a higher level (e.g., from mild to moderate or severe) at the end of treatment, it was considered to be no response, otherwise response. Criteria-3: The criteria counted the number of days with adequate relief from the participants’ diaries, and a participant with at least 14 days with adequate relief (adequate relief was defined as a VAS score < 3 cm in the evaluation of global symptoms) was recognized as achieving the global improvement of IBS-D symptoms which was considered as a response.

Response rate = (total number of participants - number of non-responders)/total number of participants × 100%.

##### 3.4.1.1 S-TXYF versus placebo

One trial (Chen et al., 2018) using evaluation criteria-3 was designed into this type of comparison. Its result showed that S-TXYF (granules) was better than placebo (RR = 1.53, 95% CI 1.09 to 2.16; NNT = 5, 4 weeks’ treatment) in increasing the response rate according to the global improvement of IBS-D symptoms score. The planned sensitivity and subgroup analyses were not carried out due to only one RCT was included in here.

##### 3.4.1.2 S-TXYF versus western medication

Ten trials using the evaluation criteria-1 involved this type of comparison, of which, one trial (Tang, 2017) also used evaluation criteria-2.

(a) Evaluation criteria-1

**FIGURE 4:**
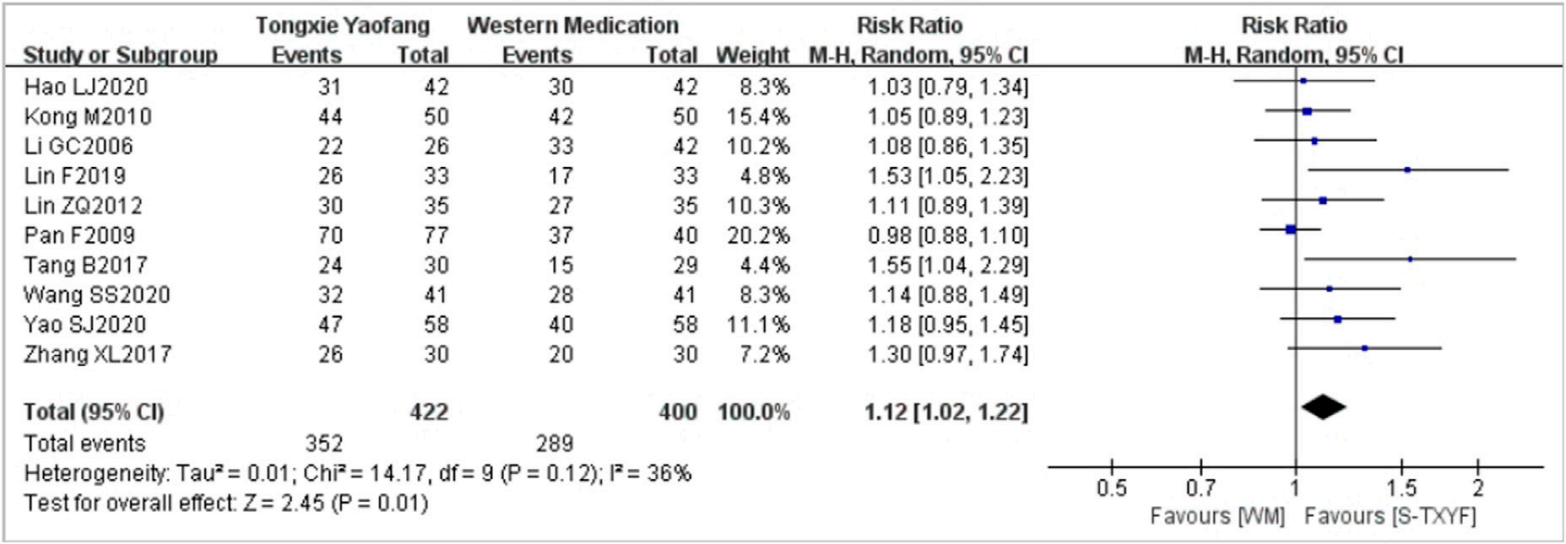
Forest plots of global improvement of IBS-D symptoms (criteria-1; random-effects).

A pooled data from 10 trials (with the treatment duration lasting between 4 weeks and 30 days) (Li, 2006; Pan et al., 2009; Kong et al., 2010; Lin, 2012; Tang, 2017; Zhang et al., 2017; Lin, 2019; Hao and Shi, 2020; Wang et al., 2020; Yao et al., 2020) showed that S-TXYF (granules and decoction) was better than western medication on this outcome but the effect difference was small (RR = 1.12, 95% CI 1.02 to 1.22, random-effects, see Figure 4; NNT = 9 (based on the pooled data); NNT of single trial is presented in Supplementary Appendix Table S2). The results of the sensitivity analysis demonstrated that there was only a slight difference between random-effects and fixed-effects (RR = 1.14, 95% CI 1.06 to 1.23, fixed-effects; Supplementary Appendix Figure S1) meta-analysis on effect sizes and both demonstrated that S-TXYF was better than western medication.

Predefined subgroup analyses according to the currently available information were conducted for the primary outcome. The forest plots of subgroup analyses are presented in Supplementary Appendix Figures S2–S4. Subgroup analysis by treatment duration did not yield a significant difference (*p* = 0.49, Supplementary Appendix Figure S2) and important effect differences between subgroups (RR = 1.10, 95% CI 0.98 to 1.22, 4 weeks, six trials; RR = 1.17, 95% CI 1.00 to 1.38, 8 weeks (1 month or 30 days), four trials; Supplementary Appendix Figure S3), but there was a statistically significant difference between trials of S-TXYF granules and S-TXYF decoction (*p* = 0.05, Supplementary Appendix Figure S3), though little effect in both groups (RR = 1.05, 95% CI 0.98 to 1.13, six trials, S-TXYF granules; RR = 1.29, 95% CI 1.06 to 1.56, four trials, S-TXYF decoction; Supplementary Appendix Figure S3). We also conducted the subgroup analysis according to the different western medications, the results (Supplementary Appendix Figure S4) showed that there was a statistically significant difference between subgroups (*p* = 0.03). S-TXYF was better than pinaverium bromide tablets (RR = 1.12, 95% CI 1.04 to 1.22, eight trials) and trimebutine maleate (RR = 1.55, 95% CI 1.04 to 2.29, one trial), but there was no statistical difference when S-TXYF compared to miyarisam (RR = 0.98, 95% CI 0.88 to 1.10, one trial).

(b) Evaluation criteria-2

One trial (Tang, 2017) used evaluation criteria-2. It showed that there was no statistical difference (RR = 1.18, 95% CI 0.83 to 1.69, NNT = 9, 8 weeks’ treatment, S-TXYF decoction) between the both groups on the response rate. We were failed to conduct the prespecified sensitivity and subgroup analyses due to only one trial was included here.

#### 3.4.2 Secondary outcomes

##### 3.4.2.1 Stool consistency

For “S-TXYF versus placebo”, one trial (Chen et al., 2018) reported stool consistency evaluated by Bristol Stool Scale after treatment. The result showed that a significantly lower Bristol Stool Score in the S-TXYF (granules) group was observed at week two than in the placebo group (5.3 ± 0.7 versus 5.6 ± 0.7; *p* = 0.02), and the effect lasted to week 12 (4.7 ± 0.8 versus 5.7 ± 0.8; *p* < 0.001).

Regarding “S-TXYF versus western medication”, three trials (Pan et al., 2009; Kong et al., 2010; Lin, 2012) reported the outcome of stool consistency and they used different tools. Of which, one trial (Lin, 2012) with a 4-week treatment showed that there was no statistical difference (MD = -0.06 points, 95% CI -0.14 to 0.02; between-group comparison of scores after treatment evaluated by Gastrointestinal Symptoms Rating Scale; S-TXYF granules), and a pooled result from the other two trials (Pan et al., 2009; Kong et al., 2010) with a 4-week treatment also showed that there was no statistical difference (MD = -0.98 points, 95% CI -2.85 to 0.90; between-group comparison of scores after treatment evaluated by the Bristol Stool Scale; *I* ^2^ = 100%) between S-TXYF (granules) and controls. An attempt to explore the source of heterogeneity by conducting subgroup analysis or sensitivity analysis was not possible due to the small number of included trials. Nevertheless, we considered that the heterogeneity may be caused by clinical factors (e.g., participants such as age and disease severity, and/or difference of comparators).

##### 3.4.2.2 Stool frequency

A total of three trials (Lin, 2012; Zhang et al., 2017; Chen et al., 2018) reported the outcome of stool frequency.

About “S-TXYF versus placebo”, one trial (Pan et al., 2009) showed that participants in S-TXYF (granules) group started to have significantly less evacuations than those in the placebo group at week 2 (13.9 ± 3.5 versus 12.7 ± 3.3 times/week; *p* = 0.01), and the effect lasted to week 12 (14.2 ± 3.0 versus 12.1 ± 3.4 times/week; *p* < 0.001).

Regarding the comparison of “S-TXYF versus western medication”, two trials (Lin, 2012; Zhang et al., 2017) evaluated this outcome. Of which, one trial (Zhang et al., 2017) with 1-month treatment showed that S-TXYF (decoction) was superior to western medication (MD = -1.56 points, 95% CI -2.26 to -0.86; between-group comparison about the difference of before and after treatment in each group) on improving the symptom, but the remaining one trial (Lin, 2012) with 4 weeks’ treatment showed that there was no statistical difference (MD = 0.01 points, 95% CI -0.11 to 0.13; between-group comparison of scores after treatment evaluated by Gastrointestinal Symptoms Rating Scale; S-TXYF granules).

##### 3.4.2.3 Abdominal pain

For “S-TXYF versus placebo”, one trial (Chen et al., 2018) reported the outcome of abdominal pain evaluated by VAS score after treatment. The trial reported that S-TXYF (granules) showed superiority over placebo in abdominal pain from week 3 (3.3 ± 1.0 versus 4.1 ± 1.1 cm; *p* < 0.001) to week 12 (3.1 ± 0.8 versus 4.2 ± 0.9 cm; *p* < 0.001).

Concerning “S-TXYF versus western medication”, four trials (Pan et al., 2009; Kong et al., 2010; Lin, 2012; Zhang et al., 2017) reported the abdominal pain evaluated by similar score scales. One trial [28] with 1-month treatment showed that S-TXYF (decoction) was superior to western medication (MD = -1.67 points, 95% CI -2.57 to -0.77; between-group comparison about the difference of before and after treatment in each group) in reducing the degree of abdominal pain. However, a pooled result from the other three trials (Pan et al., 2009; Lin, 2012; Zhang et al., 2017) with 4 weeks’ treatment showed that there was no statistical difference (MD = -0.33 points, 95% CI -1.03 to 0.37; between-group comparison of scores after treatment; *I* ^2^ = 99%) between S-TXYF (granules) and the comparator. Although large heterogeneity (*I* ^2^ = 99%) existed between the three trials (Pan et al., 2009; Kong et al., 2010; Lin, 2012; Zhang et al., 2017), we failed to carry out relevant analyses to explore the source of heterogeneity owing to the small number of included trials.

##### 3.4.2.4 Quality of life

A total of three trials (Kong et al., 2010; Tang, 2017; Yao et al., 2020) reported this outcome and all were the comparison of “S-TXYF versus western medication”.

Of these, two trials (Kong et al., 2010; Yao et al., 2020) with a 4-week treatment evaluated the outcome using different scales and showed that S-TXYF (granules) was superior to western medication in improving the quality of life (MD = 9.61 points, 95% CI 5.39 to 13.83, measured by SF-36, one trial (Kong et al., 2010); MD = 13.90 points, 95% CI 2.62 to 25.18, evaluated by a new quality of life questionnaire for patients with irritable bowel syndrome, one trial (Yao et al., 2020)). The remaining one trial (Tang, 2017) with a 8-week treatment also reported the outcome evaluated by the IBS quality of life questionnaire but failed to report the total score. The trial reported that only two dimensions of (1) interference with activity and (2) health worry in the western medication group had statistical differences before and after treatment, while S-TXYF (decoction) group had statistical differences for five dimensions: (1) dysphoria, (2) interference with activity, (3) body image, (4) health worry and (5) Food avoidance.

##### 3.4.2.5 Anxiety

Two trials (Tang, 2017; Yao et al., 2020) reported this outcome with different scales and both suggested that S-TXYF was better than western medication on relieving anxiety (MD = -4.00 points, 95% CI -6.65 to -1.35, between-group comparison of scores after treatment evaluated by SAS, S-TXYF granules, one trial (Yao et al., 2020) with 4 weeks’ treatment; MD = -2.69 points, 95% CI -5.24 to -0.14, between-group comparison of scores after treatment evaluated by Hamilton Anxiety Scale, S-TXYF decoction, one trial (Tang, 2017) with a 8-week treatment).

##### 3.4.2.6 Depression

Two trials (Tang, 2017; Yao et al., 2020) evaluated the outcome of depression by different scales. Of which, one trial (Yao et al., 2020) with a 4-week treatment suggested that S-TXYF (granules) was better than western medication on relieving depression (MD = -2.07 points, 95% CI -3.60 to -0.54, between-group comparison of scores after treatment evaluated by SDS), while the other one trial (Tang, 2017) with a 8-week treatment demonstrated that there was no statistical difference between S-TXYF (decoction) and western medication (MD = -3.58 points, 95% CI -7.76 to 0.60, between-group comparison of scores after treatment evaluated by Hamilton Depression Scale).

##### 3.4.2.7 Recurrence rate during follow-up

No trial reported the outcome of recurrence rate during follow-up.

##### 3.4.2.8 Adverse events

A total of six trials reported the outcome of adverse events.

Among the six trials: four trials (Li, 2006; Tang, 2017; Zhang et al., 2017; Lin, 2019) reported that no adverse events occurred in either the S-TXYF (granules, decoction) group or the control group; one trial (Yao et al., 2020) reported that no severe adverse events occurred in both S-TXYF (granules) and the control group. In the remaining one trial (Chen et al., 2018), 3 cases of an adverse event were occurred in the S-TXYF (granules) group (constipation, elevation in liver-enzyme and nausea after treatment) and three adverse events occurred in the S-TXYF placebo group (one case was abdominal distension and 2 cases were for nausea). The between-group difference was not significant in the rate of adverse events (*p* = 1.000).

### 3.5 Analysis of publication bias

One funnel plot was applied to explore the possibility of publication bias for trials comparing S-TXYF with western medication on the primary outcome evaluated by criteria-1, due to the number of RCTs included in the meta-analysis was more than ten.

**FIGURE 5:**
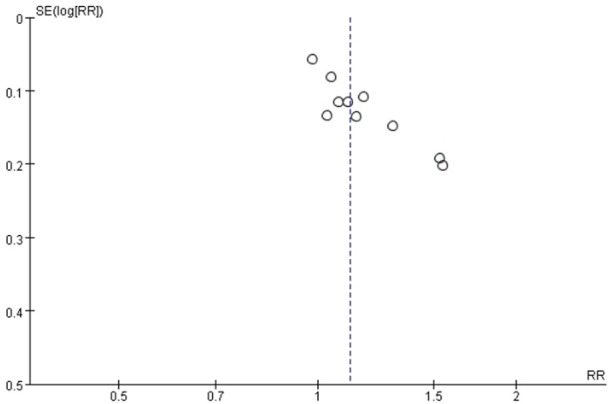
Funnel plot of exploring publication bias for trials: standard Tongxie Yaofang versus western medication on the primary outcome (criteria-1).

As can be seen from Figure 5, although the distribution of included studies was somewhat asymmetric but these were relatively concentrated and all located in the upper position, demonstrating that non-obvious publication bias probably existed.

### 3.6 Certainty of evidence (GRADE)

Certainty of the evidence for the primary outcome were all evaluated as low or moderate. They were downgraded mainly due to the high risk of performance bias (e.g., no trial achieved blinding to participants and personnel) and/or imprecision (small number of total events or small sample size). Table 2 showed the details of the certainty for the primary outcome.

**TABLE 2:** Summary of evidence certainty for the primary outcome.

Patient or population: Adult patients with diarrhea-predominant irritable bowel syndrome						
Setting: Outpatients and wards						
Experimental: Standard Tongxie Yaofang (S-TXYF)						
Control: Western medication (pinaverium bromide tablets, trimebutine maleate, miyarisam) and S-TXYF placebo granules						
Outcome: Global improvement of IBS-D symptoms						
Comparison	№ of participants (studies)	Evidence certainty (GRADE)	Anticipated absolute effects^*^ (95% CI)		Relative effect (95% CI)	Comments
			Benefit with S-TXYF	Benefit with comparator		
1. S-TXYF versus placebo (Evaluation criteria-3; 4-week treatment)	155 (1 RCT)	⊕⊕⊕○MODERATE^b^	576 per 1,000 (411–814)	377 per 1,000	RR 1.53 (1.09–2.16)	Only 1 trial (Chen et al., 2018) provided the data and no meta-analysis was carried out
2. S-TXYF versus western medication (Evaluation criteria-1; 4-week to 30-day treatment)	822 (10 RCTs)	⊕⊕⊕○MODERATE^a^	809 per 1,000 (737–881)	723 per 1,000	RR 1.12 (1.02–1.22)	None
3. S-TXYF versus western medication (Evaluation criteria-2; 8-week treatment)	59 (1 RCT)	⊕⊕○○LOW^a,b^	732 per 1,000 (515–1,000)	621 per 1,000	RR 1.18 (0.83–1.69)	Only 1 trial (Tang, 2017) provided the data and no meta-analysis was carried out

The reasons for the downgrade of evidence: a. high risk of performance bias (e.g., no trial achieved blinding to participants and personnel); b. imprecision (small number of total events or small sample size).

CI: confidence interval; RR: risk ratio; RCT: randomized controlled trial.

Global improvement of IBS-D symptoms: measured by a validated scale or efficacy evaluation criteria, such as IBS severity scoring system (IBS-SSS), or other scales or criteria with a clear description.

*The risk in the intervention group (and its 95% confidence interval) is based on the assumed risk in the comparison group and the relative effect of the intervention (and its 95% CI).

GRADE Working Group grades of evidence.

High certainty: We are very confident that the true effect lies close to that of the estimate of the effect.

Moderate certainty: We are moderately confident in the effect estimate: The true effect is likely to be close to the estimate of the effect, but there is a possibility that it is substantially different.

Low certainty: Our confidence in the effect estimate is limited: The true effect may be substantially different from the estimate of the effect.

Very low certainty: We have very little confidence in the effect estimate: The true effect is likely to be substantially different from the estimate of effect.

## 4 Discussion

### 4.1 Summary of main findings

A total of 11 RCTs which evaluated the therapeutic effects of S-TXYF for IBS-D were included. Regarding the global improvement of IBS-D symptoms, S-TXYF was superior to western medication and placebo. In relation to the secondary outcome, S-TXYF was better than placebo in the improvement of stool consistency, stool frequency and abdominal pain, as well as was superior to western medication on improving the quality of life and relieving anxiety.

Six trials mentioned the occurrence of adverse events. Of them, five trials reported that no occurrence of (severe) adverse event in either group; and one trial reported that 3 cases with adverse events occurred in the S-TXYF group and 3 cases with adverse events occurred in placebo group. Therefore, the use of S-TXYF appears to be safe as there was no difference when compared to the placebo and western medication for the occurrence of adverse events.

### 4.2 Comparison with previous research

A recently published overview of systematic reviews and meta-analyses (Zhou et al., 2019) showed 10 previous researches on modified-TXYF (or and S-TXYF) for IBS-D. The modified-TXYF is defined as a limited number of additional Chinese herbal medicines being added to S-TXYF according to the different symptoms of participants with individualized treatment (syndrome differentiation). However, the clinical evidence on modified-TXYF has some limitations for clinical guidance, e.g., the research is difficult to repeat and the treatment regimen varies due to the pragmatic nature of clinical practice. Since the complexity of modified-TXYF also makes standardization of this herbal product difficult [*Which does not mean that syndrome differentiation or modified-TXYF is not desirable in clinical*]*,* we included RCTs using S-TXYF only. In addition, we further specifically retrieved the systematic reviews and meta-analyses of IBS-D treated with only S-TXYF, but no relevant review was found.

Therefore, in our pinion, this is the first systematic review and meta-analysis based on RCTs to evaluate the therapeutic effects and safety of S-TXYF for IBS-D.

### 4.3 Implications for future clinical practice

What we can tell from the results is that S-TXYF can bring even more benefits in terms of the global improvement of IBS-D symptoms, reducing the abdominal pain of patients with IBS-D, improving the single symptom of stool consistency and stool frequency compared to placebo. Although there was no statistical difference between S-TXYF and western medication on improving stool consistency, S-TXYF had more potential for increasing the response rate of global improvement of IBS-D symptoms, improving the quality of life and alleviating anxiety. Moreover, the use of S-TXYF appears to be safe as there was no difference with placebo and western medication in the occurrence of adverse events. In summary, S-TXYF has potential in the treatment of IBS-D and may be as a selection to treat IBS-D.

From the characteristics of included RCTs, treatment duration varied from 4 to 8 weeks (30 days or 1 month). Which suggests that a treatment duration of no less than 4 weeks for S-TXYF in the treatment IBS-D is needed. Of course, whether less than 4 weeks of treatment is effective needs further research. Regarding the doses of the S-TXYF granules or the S-TXYF decoction, as well as the doses of the four herbal medicines (*Rhizoma Atractylodis Macrocephalae., Paeoniae Radix Alba, Citri Reticulatae Pericarpium* and *Saposhnikoviae Radix*) or the four herbal medicines' extract, we could not give a recommendation as the dose of them was varied in different trials. The differences of dose ratios of the four herbal medicines also exist in the different ancient books e.g. Danxi Xinfa (Yuan Dynasty, 1271-1638; the ratio of *Rhizoma Atractylodis Macrocephalae., Paeoniae Radix Alba, Citri Reticulatae Pericarpium* and Saposhnikoviae Radix was “3:2:1.5:1”), Yixue Zhengzhuan (Ming Dynasty, 1368-1644; “2:2:1.5:1”), Jingyue Quanshu (Ming Dynasty; “3:2:1.5:2”), etc. (Chu, 2006). But with the aim of giving more references to the clinical practice, we reported the relevant information regarding the doses of the four herbal medicines from all the included trials in Table 1. From subgroup analysis results of the primary outcome and analysis results of secondary outcomes, although the form of the product seems to have little effect on the therapeutic effects, most outcomes were statistically different when S-TXYF decoction was compared with western medication. Previous publications (Zhu et al., 2020; Jia and Du, 2021) have also proposed that the treatment of functional gastrointestinal diseases with integrated Chinese and western medicine may have more advantages than traditional Chinese medicine or western medication alone. Therefore, clinicians may try to use S-TXYF combined with western medication recommended by guidelines (e.g. pinaverium bromide tablets, Trimebutine Maleate) to treat IBS-D, but the therapeutic effects and safety need to be supported by further research evidence.

### 4.4 Implications for future clinical trials

The risk bias of one trial was considered as "low risk of bias" and the remaining trials were judged to be at “high risk of bias” for the trial design, which suggests that most of them require some improvement. Ten out of 11 trials did not achieved blinding for participants, researchers and assessors, which could lead to outcomes being biased by participants, researchers and assessors beliefs compared to blinded trials (Flower et al., 2014). Only one trial (1/11) reported information about the trial protocol. It is recommended that future trials should be designed with reference to the relevant items mentioned in the Standard Protocol Items: Recommendations for Interventional Trials (SPIRIT) guidelines (Chan et al., 2013) and ROB 2.0 (Sterne et al., 2019), and the trial protocol should be formulated and registered in advance. In addition, the researcher should report their trial following the Consolidated Standards of Reporting Trials (CONSORT) statement (Schulz et al., 2010).

As the mention previously, the dose ratio of *Rhizoma Atractylodis Macrocephalae., Paeoniae Radix Alba, Citri Reticulatae Pericarpium* and *Saposhnikoviae Radix* was varied in different trials as well as in the different ancient books. However, in the treatment of diseases with traditional Chinese medicine, the dose (ratio) of Chinese herbal medicines of each treatment formulae is one of the key factors affecting the therapeutic effects. Therefore, it is necessary to confirm the acceptable range of different Chinese herbal medicines’ doses and proportions in S-TXYF to achieve the best therapeutic effects of S-TYXF against IBS-D. So it could be an area for future research work.

With regard to the evaluation on the therapeutic effects of S-TXYF, it is recommended that future trials should to pay more attention on the evaluation of the outcomes that are closely related to IBS-D with approved evaluation tools. The main consideration to suggest this point was that most included trials did not report the outcomes of symptoms that closely related to IBS-D, such as stool consistency, stool frequency, abdominal pain. Furthermore, no trial reported recurrence rate during the follow-up after the end of treatment to focus on the long-term therapeutic effects of S-TXYF in the treatment of IBS-D. Which suggests that future research should also pay attention to the outcome of long-term benefits.

Although our research demonstrated that there was a statistically significant difference on some outcomes between S-TXYF and western medication, the difference were little and probably did not show clinical significance. Therefore, the evaluation of cost-benefit may be particularly important, but none of the included trials reported data on this aspect. So it is suggested that future trials should also focus on the evaluation and reporting of cost-benefit, as this will be more helpful for medical decision-making.

### 4.5 Strengths and limitations

Our review not only confirmed the global improvement of IBS-D symptoms, but also on the improvement of specific symptoms that are closely related to IBS-D. To our knowledge, this is the first systematic review to evaluate the therapeutic effects and safety of S-TXYF alone in the treatment of IBS-D. Furthermore, certainty of the evidence for the primary outcome was assessed using the GRADE approach. For the dichotomous outcomes, we also calculated the NNT value to provide a more intuitive reference for clinical practice. Therefore, the evidence strengthens the therapeutic value of S-TXYF for future clinical practice and study.

One of the limitations for our review is the small number of included RCTs, especially RCTs regarding “S-TXYF versus placebo”. However, which also suggests that more relevant RCTs should be carried out in the future.

## 5 Conclusion

Although current results showed that S-TXYF may have potential to treat IBS-D and its use appears to be safe, no a clear and confirmed conclusion can be drawn from our review as the overall inadequate design of the included trials reviewed. So more rigorous trials are warranted to establish confirmed evidence on its benefits and safety.

## Data Availability

The original contributions presented in the study are included in the article/Supplementary Material further inquiries can be directed to the corresponding author.

## Abbreviations

AE, Adverse events; CI, Confidence intervals; IBS-D, Diarrhea-predominant irritable bowel syndrome; IBS-SSS, IBS Severity Scoring System; IBS-QOL, a new quality of life questionnaire for patients with irritable bowel syndrome; MD, Mean difference; RCT, Randomized controlled trial; RR, Risk ratio; SF-36, the MOS 36-item short-from health survey; SAS, the self-rating anxiety scale; SDS, the self-rating depression scale; S-TXYF, standard Tongxie Yaofang.
