# Supplementary figures and images for: Systematic review and meta-analysis of Chinese herbal formula Tongxie Yaofang for diarrhea-predominant irritable bowel syndrome: Evidence for clinical practice and future trials
Source: Front Pharmacol. 2022 Aug 25;13:904657. doi: 10.3389/fphar.2022.904657 (PMC9452967; doi:10.3389/fphar.2022.904657)

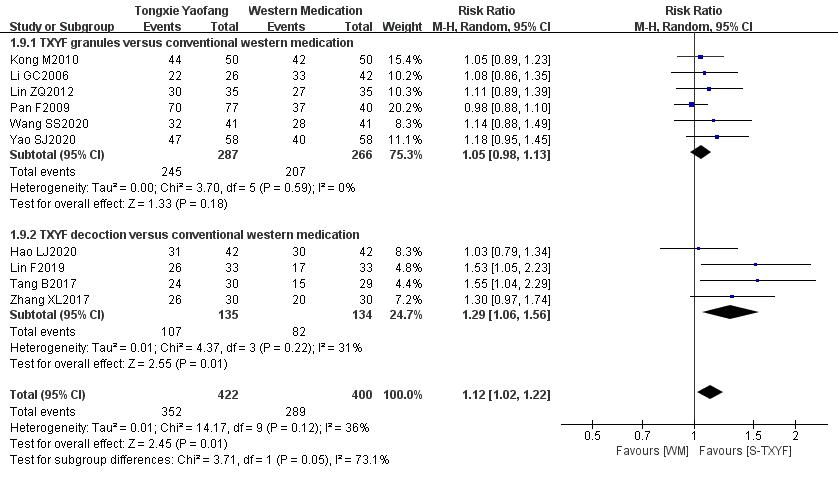

Supplement: Supplementary file 1 [file Image3.jpg]

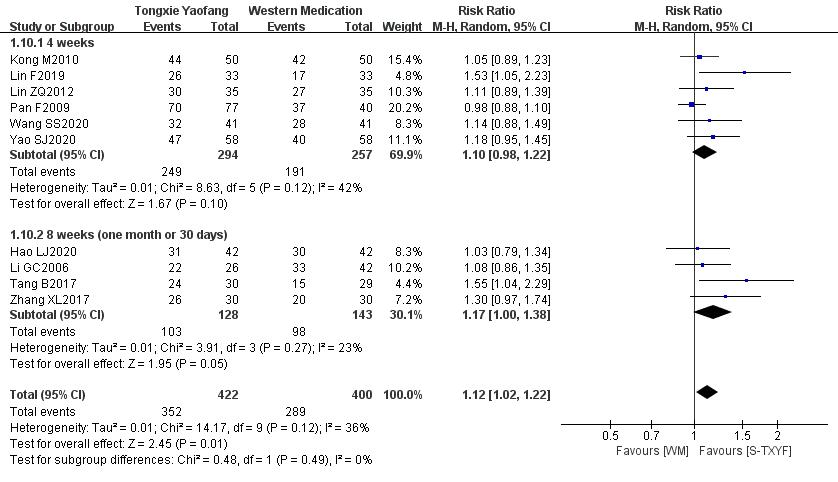

Supplement: Supplementary file 2 [file Image2.jpg]

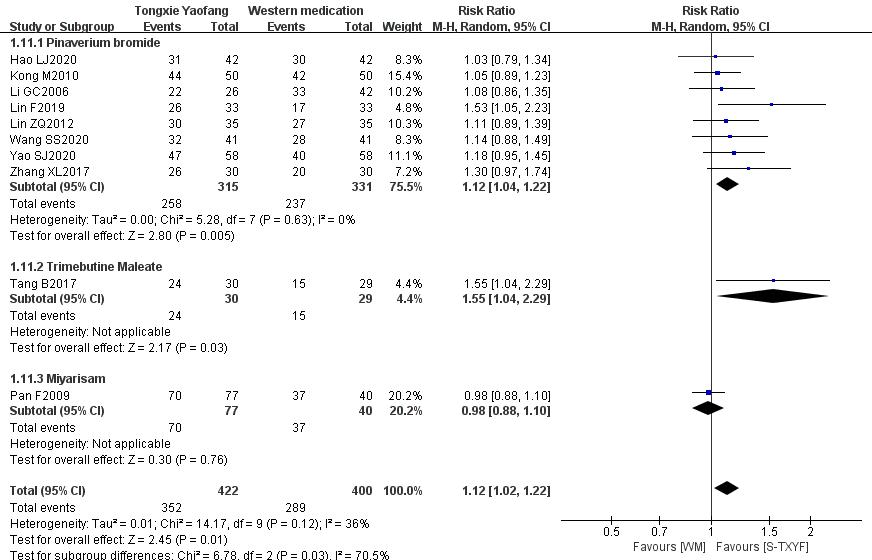

Supplement: Supplementary file 5 [file Image4.jpg]

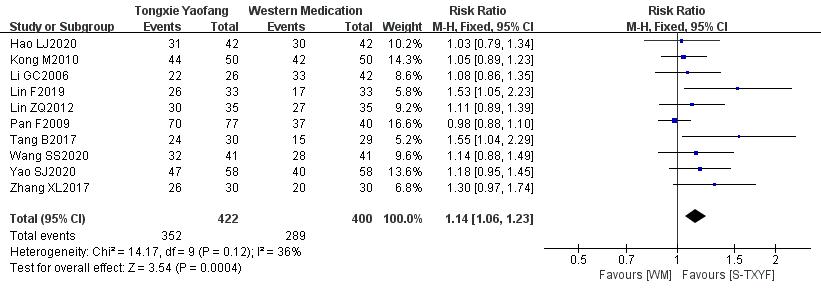

Supplement: Supplementary file 6 [file Image1.jpg]
