# Supplementary material for: Systematic review and meta-analysis of Chinese herbal formula Tongxie Yaofang for diarrhea-predominant irritable bowel syndrome: Evidence for clinical practice and future trials
Source: Front Pharmacol. 2022 Aug 25;13:904657. doi: 10.3389/fphar.2022.904657 (PMC9452967; doi:10.3389/fphar.2022.904657)
Supplement: Supplementary file 3 [file Table1.docx]

**Appendix-Table 1 Search strategies for each databases**

| **Database** | **Search strategy** |
| --- | --- |
| PubMed | (((((TongxieYaofang[Title/Abstract]) OR (Tong Xie Yao Fang[Title/Abstract])) OR (Tong-Xie-Yao-Fang[Title/Abstract])) OR (TXYF[Title/Abstract])) AND ((Irritable bowel syndrome[Title/Abstract]) OR (IBS[Title/Abstract]))) |
| the Cochrane Library | (TongxieYaofang OR Tong Xie Yao Fang OR TXYF OR Tong-Xie-Yao-Fang) AND (Irritable bowel syndrome OR IBS) in Title Abstract Keyword - (Word variations have been searched) |
| Embase | #1: tongxieyaofang:ti,ab,kw OR 'tong xie yao fang':ti,ab,kw OR txyf:ti,ab,kw  #2: 'irritable bowel syndrome':ti,ab,kw OR 'intestinal irritable syndrome':ti,ab,kw OR 'the irritable bowel syndrome':ti,ab,kw OR ibs:ti,ab,kw  #3: #1 AND #2 |
| Web of Science | TS=(TongxieYaofang OR Tong Xie Yao Fang OR Tong-Xie-Yao-Fang OR TXYF) AND TS = (Irritable bowel syndrome OR IBS) |
| SinoMed | ( "痛泻要方"[标题]) AND ("腹泻型肠易激综合征"[标题] OR "IBS-D"[标题]) |
| the Chinese National Knowledge Infrastructure Databases  (CNKI) | ((SU %= '痛泻要方') AND (SU %= '腹泻型肠易激综合征' OR SU % = 'IBS-D')) |
| Wanfang Database | (主题:(痛泻要方) and (主题:(腹泻型肠易激综合征) or 主题:(IBS-D))) |
| the Chongqing Chinese Science and Technology Journal Database  (VIP) | ((M = 痛泻要方) AND (M = 腹泻型肠易激综合征 OR M = IBS-D)) |
