# Supplementary material for: Systematic review and meta-analysis of Chinese herbal formula Tongxie Yaofang for diarrhea-predominant irritable bowel syndrome: Evidence for clinical practice and future trials
Source: Front Pharmacol. 2022 Aug 25;13:904657. doi: 10.3389/fphar.2022.904657 (PMC9452967; doi:10.3389/fphar.2022.904657)
Supplement: Supplementary file 4 [file Table2.docx]

**Appendix Table S2 NNT of per single trial for the Primary outcome (Global improvement of IBS-D symptoms: S-TXYF versus western medication, Evaluation criteria-1)**

| **No.** | **Study ID** | **NNT** | **Treatment duration** | **Comment** |
| --- | --- | --- | --- | --- |
| 1 | Hao et al., 2020 | 42 | 8 weeks | For every 42 patients treated, **1 more** patient with the global improvement of IBS-D symptoms occurred in the S-TXYF group than in the control group. |
| 2 | Kong et al., 2010 | 25 | 4 weeks | For every 25 patients treated, **1 more** patient with the global improvement of IBS-D symptoms occurred in the S-TXYF group than in the control group. |
| 3 | Li, 2006 | 17 | 30 days | For every 17 patients treated, **1 more** patient with the global improvement of IBS-D symptoms occurred in the S-TXYF group than in the control group. |
| 4 | Lin, 2019 | 4 | 4 weeks | For every 4 patients treated, **1 more** patient with the global improvement of IBS-D symptoms occurred in the S-TXYF group than in the control group. |
| 5 | Lin, 2012 | 12 | 4 weeks | For every 12 patients treated, **1 more** patient with the global improvement of IBS-D symptoms occurred in the S-TXYF group than in the control group. |
| 6 | Pan et al., 2009 | 63^▼^ | 4 weeks | For every 63 patients treated, **1 less** patient with the global improvement of IBS-D symptoms occurred in the S-TXYF group than in the control group. |
| 7 | Tang, 2017 | 4 | 8 weeks | For every 4 patients treated, **1 more** patient with the global improvement of IBS-D symptoms occurred in the S-TXYF group than in the control group. |
| 8 | Wang et al., 2020 | 11 | 4 weeks | For every 11 patients treated, **1 more** patient with the global improvement of IBS-D symptoms occurred in the S-TXYF group than in the control group. |
| 9 | Yao et al., 2020 | 9 | 4 weeks | For every 9 patients treated, **1 more** patient with the global improvement of IBS-D symptoms occurred in the S-TXYF group than in the control group. |
| 10 | Zhang et al., 2017 | 5 | 1 month | For every 5 patients treated, **1 more** patient with the global improvement of IBS-D symptoms occurred in the S-TXYF group than in the control group. |
